# What determines the neural response to snakes in the infant brain? A systematic comparison of color and grayscale stimuli

**DOI:** 10.3389/fpsyg.2023.1027872

**Published:** 2023-03-13

**Authors:** Julie Bertels, Adelaïde de Heering, Mathieu Bourguignon, Axel Cleeremans, Arnaud Destrebecqz

**Affiliations:** ^1^ULBabyLab, Consciousness, Cognition and Computation Group (CO3), Center for Research in Cognition and Neurosciences (CRCN), ULB Neuroscience Institute (UNI), Université Libre de Bruxelles (ULB), Brussels, Belgium; ^2^Laboratoire de Neuroanatomie et de Neuroimagerie Translationnelles (LN^2^T), ULB Neuroscience Institute (UNI), Université Libre de Bruxelles (ULB), Brussels, Belgium; ^3^LulLABy, Unité de Recherche en Neurosciences Cognitives (UNESCOG), Center for Research in Cognition and Neurosciences (CRCN), ULB Neuroscience Institute (UNI), Université Libre de Bruxelles (ULB), Brussels, Belgium; ^4^Laboratory of Neurophysiology and Movement Biomechanics, ULB Neuroscience Institute (UNI), Université Libre de Bruxelles (ULB), Brussels, Belgium

**Keywords:** infancy, snakes, steady-state visual evoked potential, color, EEG

## Abstract

Snakes and primates have coexisted for thousands of years. Given that snakes are the first of the major primate predators, natural selection may have favored primates whose snake detection abilities allowed for better defensive behavior. Aligning with this idea, we recently provided evidence for an inborn mechanism anchored in the human brain that promptly detects snakes, based on their characteristic visual features. What are the critical visual features driving human neural responses to snakes is an unresolved issue. While their prototypical curvilinear coiled shape seems of major importance, it remains possible that the brain responds to a blend of other visual features. Coloration, in particular, might be of major importance, as it has been shown to act as a powerful aposematic signal. Here, we specifically examine whether color impacts snake-specific responses in the naive, immature infant brain. For this purpose, we recorded the brain activity of 6-to 11-month-old infants using electroencephalography (EEG), while they watched sequences of color or grayscale animal pictures flickering at a periodic rate. We showed that glancing at colored and grayscale snakes generated specific neural responses in the occipital region of the brain. Color did not exert a major influence on the infant brain response but strongly increased the attention devoted to the visual streams. Remarkably, age predicted the strength of the snake-specific response. These results highlight that the expression of the brain-anchored reaction to coiled snakes bears on the refinement of the visual system.

## Introduction

Snakes and primates have coexisted for thousands of years. Given that snakes are the first of the major primate predators (Isbell, 2009), natural selection may have favored primates with appropriate defensive behavior that increased their chances of survival. Such defensive behavior requires quick and efficient prior detection of the danger, and many studies have indeed demonstrated that human and non-human primates developed, accordingly, the propensity to rapidly detect snake-like visual cues. As a consequence, they detect snakes faster than non-snakes in a collection of pictures (Soares et al., 2014; Kawai and Koda, 2016). This predisposition is functional early in development, sensitive to snake characteristic features such as their coiled aspect, and subtended by a neurobiological substrate (Bertels et al., 2020), as evidenced notably by the existence of thalamic neurons in the macaque brain that selectively respond to snake pictures (Van Le et al., 2013).

Several studies support the idea of an inborn predisposition. As a matter of fact, human infants, who have no idea of how dangerous snakes can be and have never experienced these animals, are remarkable snake detectors, like their older peers and non-human primates (Deloache and Lobue, 2009; LoBue and DeLoache, 2010; Bertels et al., 2018). Recently, we provided electrophysiological evidence that glancing at snakes engenders specific occipital responses in the infant brain (Bertels et al., 2020). Indeed, we recorded the brain electrical activity of 7- to 10-month-olds when they watched a series of animal pictures flickering at 6 Hz. Depending on the sequences, snake, frog, or caterpillar images appeared every five images (i.e., at 1.2 Hz). We observed a snake-specific neural response at 1.2 Hz and its harmonics, which was larger in amplitude than that generated by frogs or caterpillars. These results support that humans are, very early on, equipped with a brain-anchored mechanism sensitive to snake prototypical features, functional from the first months of life and independent of any prior exposure to snakes.

What are the critical features driving that response to snakes in infants, and more generally in primates, is an unresolved issue. Their prototypical curvilinear coiled shape is of major importance (e.g., Lobue and Deloache, 2011; Van Strien et al., 2016; Gomes et al., 2018). Indeed, caterpillars, which are elongated as snakes, but not coiled, do not elicit any specific brain response in the occipital brain areas (Bertels et al., 2020). Nevertheless, it could still be that the infant brain responds to a mix of snake-like physical traits (Kawai, 2019), including their scale patterns (Isbell and Etting, 2017; Van Strien and Isbell, 2017), striking posture (Masataka et al., 2010) and coloration (Masataka et al., 2010; Hayakawa et al., 2011). These traits were mixed up in Bertels et al. (2020). In the present study, we aim at specifically examining whether, in human infants, color affects the neural response to coiled snakes.

Color information is a critical cue when processing natural scenes, for several reasons. When applied to images, color is not only capturing attention more than grayscale variations (Zhu et al., 2013) but it also facilitates their segmentation from the background (Delorme et al., 1999; Gegenfurtner and Rieger, 2000), eases discrimination between different categories of stimuli, and contributes to the generalization of variant exemplars of the same category (Or et al., 2019). Accordingly, the importance of color cues for recognition and categorization of natural scenes has already been demonstrated in adults (Gegenfurtner and Rieger, 2000; Oliva and Schyns, 2000; Goffaux et al., 2005). At a neural level, Goffaux et al. (2005) evidenced, for example, that chromatic information speeds up early scene categorization. More recently, Or et al. (2019) showed that color contributes to the rapid detection of faces among natural images, eliciting larger brain responses over occipitotemporal areas than grayscale face pictures.

Regarding snake detection, color information might be especially important. Although snakes do not share a single diagnostic color, their bright, conspicuous coloration could act as an aposematic signal, serving to repel predators and warn preys (Ruxton et al., 2004; Stevens and Ruxton, 2012; Souchet and Aubret, 2016; Prokop et al., 2018). Also, snakes’ coloration might, in conjunction with their diagnostic shape, act as a trigger for the rapid detection and the fear responses in primates (Souchet and Aubret, 2016). However, the exact contribution of color cues to snake detection is mixed. Although children detect aposematically colored snakes faster than cryptically colored snakes (Hayakawa et al., 2011; Fančovičová et al., 2020), their rapid detection also operates for grayscale pictures in children, adults, and monkeys (e.g., Shibasaki and Kawai, 2009; Masataka et al., 2010; Hayakawa et al., 2011; Lobue and Deloache, 2011; He et al., 2014). Color would therefore not be necessary for children to rapidly detect snakes. In infants, the question remains unsolved. As they are naïve subjects with immature visual systems and who have not yet learned to fear these reptiles, they could especially benefit from combined visual cues, including color, being indicative of danger.

The present study investigates whether color information contributes to the specific neural response to snakes in the infant brain, using fast periodic visual stimulation combined with electroencephalography, in trichromat infants, i.e., who discriminate colors in the green-red part of the spectrum. We first aimed at replicating Bertels et al.’s (2020) findings of a specific response to fleeting pictures of snakes in their natural background, in comparison to threat-irrelevant creatures. We then assessed how the colorful nature of the stimuli impacted these responses, and finally evaluated to what extent these responses develop with age.

## Methods

### Participants

Twenty-two full-term infants (6–11 months old; mean ± SD age, 271 ± 43 days; 9 males) with no known neurodevelopmental disorder were included in the study. Infants from the color group (*n* = 11) were presented with the color versions of the pictures, while those of the grayscale group (*n* = 11) were presented with their grayscale versions. Questions to the parents revealed they were not especially familiar with snakes or frogs. The parents gave informed consent prior to testing. The CUB Hôpital Erasme Ethics Committee approved the experimental protocol. The experiments were carried out in accordance with the approved guidelines and regulations.

### Stimuli and procedure

**Figure 1:**
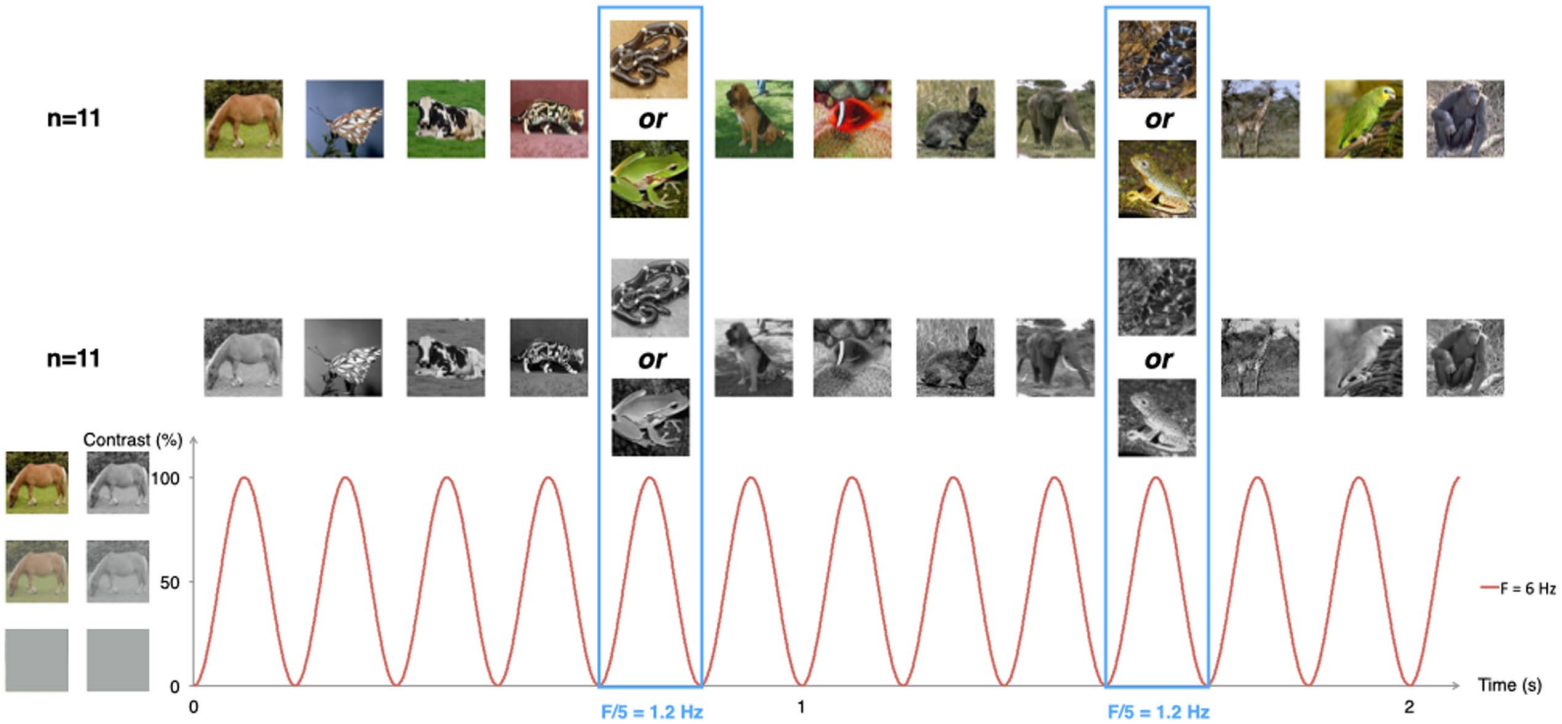
Schematic illustration of the experimental paradigm. Animal pictures were presented by sinusoidal contrast modulation at a rate of 6 per second (*F* = 6 Hz). Snake or frog pictures were presented every fifth stimulus (*F* = 6/5 = 1.2 Hz), in different trial sequences. Half of the infants were presented with color pictures, the other half with grayscale versions of the same pictures.

Infants were presented with pictures of animals in their natural habitat, from various angles, as in Bertels et al. (2020) (see Figure 1). Infants viewed either color or grayscale versions of the pictures. Both sets were equalized in terms of luminance and contrast using Matlab (Mathworks, United States) and further resized to 200 × 200 pixels.

Snake and frog pictures (29 exemplars of each category) were taken from a set of pictures from LoBue and DeLoache (2008). Snakes were all depicted coiled, none in an attack posture. Frogs were used as non-snake control stimuli since they resemble snakes in texture, brightness, and color, and since they are as unfamiliar to infants as snakes are (Lobue and DeLoache, 2008). Other animal pictures were taken from a set of 130 pictures of 13 different species.^1^

Pictures were presented centrally on a 800 × 600 pixels resolution monitor, on a light grey background. Monitor refreshment rate was 60 Hz. Viewed from 40 cm of distance, images spanned a visual angle of ~13 × 13 degrees.

Visual stimulation was performed using the Psychtoolbox 3.0.9 software for Windows running on Matlab 7.6 (MathWorks Inc.). Images appeared on the screen within 24-s sequences, including a 2-s fade-in and a 2-s fade-out. There was a total of 144 different images per sequence. Each image lasted for 166.7 ms, yielding a presentation rate of 6 Hz (stimulation frequency). The intensity at every pixel was modulated by a squared-sine wave with null contrast at the beginning and end of stimulus presentation and reaching maximal contrast at 83.35 ms (see Figure 1).

Sequences were built as alternations of 4 non-frog non-snake animal pictures followed by 1 frog or snake picture, in frog and snake sequences, respectively. Half of the infants within each group started with a snake sequence. In this setting, the repetition rate of frog or snake images was therefore of 1.2 Hz (6 Hz/5), hereafter referred to as the category-selective frequency. Discrimination by the infant’s visual system of frogs or snakes would therefore lead to the identification, in the EEG spectrum, of a response at 1.2 Hz.

Infants were tested in a dimly lit and quiet room. They were sitting in a car seat, with a caregiver behind. The experimenter monitored their looking behavior with a webcam and started each sequence once the infant looked at the screen. Infants watched as many sequences as they could (*M* = 13, SD = 2.8, range = 9–17 in the color group; *M* = 8, SD = 2.72, range 5–12 in the grayscale group). Testing lasted 3–10 min.

### Electroencephalography acquisition

Electroencephalography recordings were obtained using a 32-channel BioSemi Active2 system (Amsterdam, Netherlands), with electrodes including standard 10–20 system locations and 2 additional reference electrodes. Signal was recorded at 1024 Hz. The offset of each electrode was kept between ± 25 𝜇V by injecting each electrode with saline gel.

Triggers were used to indicate, at the beginning of each sequence, whether infants viewed a frog or a snake sequence. They were also sent at each image presentation, when its contrast was of 0%.

### Electroencephalography pre-processing

To pre-process EEG signals, we used Letswave 6^2^ running on MATLAB R2017a (Mathworks Inc.), and followed a standard procedure (e.g., Liu-Shuang et al., 2014).

Individual EEG signals were first band-pass filtered (0.1–100 Hz) and resampled to 250 Hz. Trials of 28 s were extracted from the continuous data, from 2 s before sequence onset to 2 s after sequence offset (see, e.g., de Heering and Rossion, 2015). Trials were further examined for possible channel artifacts. Noisy channels (maximum 3 per infant) were reconstructed by linear interpolation of 2 surrounding clean channels. This was done for six infants. EEG signals were then re-referenced to their common average, and trimmed to exclude the fade-in and fade-out. This resulted in 20-s stimulation sequences.

We then applied a Fast Fourier Transform (FFT) to these 20-s sequences (frequency resolution, 1/20s = 0.05 Hz). The Fourier coefficients of each sequence were further converted into signal-to-noise ratios (SNRs) by taking the ratio between the amplitude at a frequency bin of interest and the mean amplitude at the 12 surrounding frequency bins (6 on each side, excluding the immediately adjacent bins, see Peykarjou et al., 2017; Barry-Anwar et al., 2018).

Given that the infant brain should not synchronize to the stimulation frequency if they are not watching the pictures, sequences with an SNR below 2 at 6 Hz (stimulation frequency) at all the medial occipital electrodes (O1, O2, and Oz) were discarded (see de Heering and Rossion, 2015; Peykarjou et al., 2017; Bertels et al., 2020). This step led to rejecting 2.55 ± 2.36 (mean ± SD; range, 0–7) sequences per infant. The number of sequences kept in the frog (4.14 ± 1.75; range 1–8) and snake condition (3.95 ± 1.86; range 1–8) did not differ significantly (*p* > 0.40). The number of sequences retained was 87 for snake (55 color and 32 grayscale) and 91 for frog sequences (58 color and 33 grayscale). Four frog sequences (3 color and 1 grayscale) were also randomly discarded to ensure comparability of SNR measures between conditions.

### Frequency domain analyses

Custom-made MATLAB scripts were used for the analyses. For each remaining sequence, corresponding Fourier coefficients were divided by a single normalization factor taken as the mean amplitude of the Fourier coefficients for frequencies within 0.6–1.8 Hz, a frequency range that surrounded the category-selective frequency (1.2 Hz). This procedure ensured all sequences were given about the same weight at 1.2 Hz, even when they contained excessive movement artifacts. To restore the usual units and scales of the Fourier coefficients, they were all multiplied by the median across sequences of the normalization factor. Raw amplitude spectra were obtained for each condition and electrode as the modulus of the averaged sequences of Fourier coefficients, at the subject level and the group level (averaging the 55 color sequences, and the 32 grayscale sequences, disregarding their origin in terms of participants’ identity). These raw spectra at each channel were further corrected by subtracting from the amplitude at each frequency bin, that averaged across the 12 surrounding bins (see Rekow et al., 2021; Supplementary Figure S1). In addition, raw amplitude spectra were converted to SNR responses as described above. SNR responses were further averaged across the 3 occipital electrodes (O1, O2, and Oz) where the responses were observed in Bertels et al. (2020).

In view of statistical appraisal, we also estimated *Z*-scores at the group and the individual level. *Z*-scores were obtained as the difference between amplitude at each frequency bin and mean amplitude at the 12 surrounding frequency bins (excluding the immediately adjacent bins, see below) divided by the standard deviation of the amplitude at these 12 surrounding bins. We hypothesized that stronger responses will be generated at 1.2 Hz and harmonics, for snake than frog pictures.

### Statistics

We tested the statistical significance of responses at category-selective frequency (1.2 Hz) and harmonics under 12 Hz (i.e., 1.2, 2.4, 3.6, 4.8, 7.2, 8.4, 9.6, and 10.8 Hz, see Retter et al., 2021). The significance of category-selective responses in infants indeed never exceeds the first harmonics (Peykarjou, 2022). To do so, we used a statistical test akin to a permutation test (Nichols and Holmes, 2002). The test was applied to both group-and subject-level responses for frog and snake sequences separately. The null hypothesis under testing was that frog and snake stimuli would elicit responses of similar amplitude as non-frog non-snake stimuli, leading to a *Z*-score at 1.2 Hz (and harmonics) that does not depart significantly from values expected by chance. To test this hypothesis, we used a previously published test specifically designed to overcome the difficulty linked to the fact that a single *Z*-score was obtained for all participants’ data. The test was applied to amplitude at O1, Oz and O2, considered here as the channels of interest (Bertels et al., 2020). The starting point was to re-estimate the *Z*-scores at each tested frequency, averaged across the three occipital electrodes, based on sequences in which either the first or last cycle was removed. In that framework, a permutation distribution (1,000 permutations) for that *Z*-score was built from sequences trimmed in a way that randomizes the position of the frog or snake images while preserving synchrony in image presentation. Namely, for each permutation, we removed the data corresponding to the *n* first images and 5–*n* last images, *n* being a random integer between 0 and 4. With this approach, the phase-locking of possible responses specific to frog or snake images was disrupted. We considered the proportion of values in the permutation distribution that were above the observed value is a robust statistical estimate of response (and *Z*-score) significance.

When comparing the SNR between groups or conditions, it is generally statistically advantageous to consider the SNR response averaged across multiple harmonics. This approach is well grounded given that multiple harmonics do not have a direct meaning in terms of underlying pathophysiological processes (Heinrich, 2010; Norcia et al., 2015). In adults, a common approach is to consider all harmonics before the first non-significant one (e.g., Liu-Shuang et al., 2014). However, in infants, responses are usually confined to fewer harmonics (< 3, maximum 9, see Peykarjou, 2022, for a systematic review) that are not necessarily subsequent (e.g., Peykarjou et al., 2022). Therefore, in a second step, we considered the average of the responses across the successive harmonics of 1.2 Hz until the last significant response (i.e., until 7.2 Hz, see “Results” section). This selection of harmonics is warranted by the fact that considering non-significant harmonic responses has no detrimental effect on the quantification of the response (Rossion et al., 2020; Peykarjou et al., 2022).

To compare group-level responses between frog and snake sequences, separately at category-selective and stimulation (6 and 12 Hz) frequencies, we also used a statistical test akin to a permutation test (see above). In that test, the *Z*-scores were contrasted between conditions, and this contrast was compared to a permutation distribution (1,000 permutations) wherein the contrast value was obtained after having shuffled frog and snake sequences. The same procedure was used to compare group-level responses between colorful and grayscale sequences.

Finally, we used Spearman correlations to test the relationship between the infants’ age and their neural responses to snakes and frogs.

## Results

### Stimulation responses (6 and 12 Hz)

**Figure 2:**
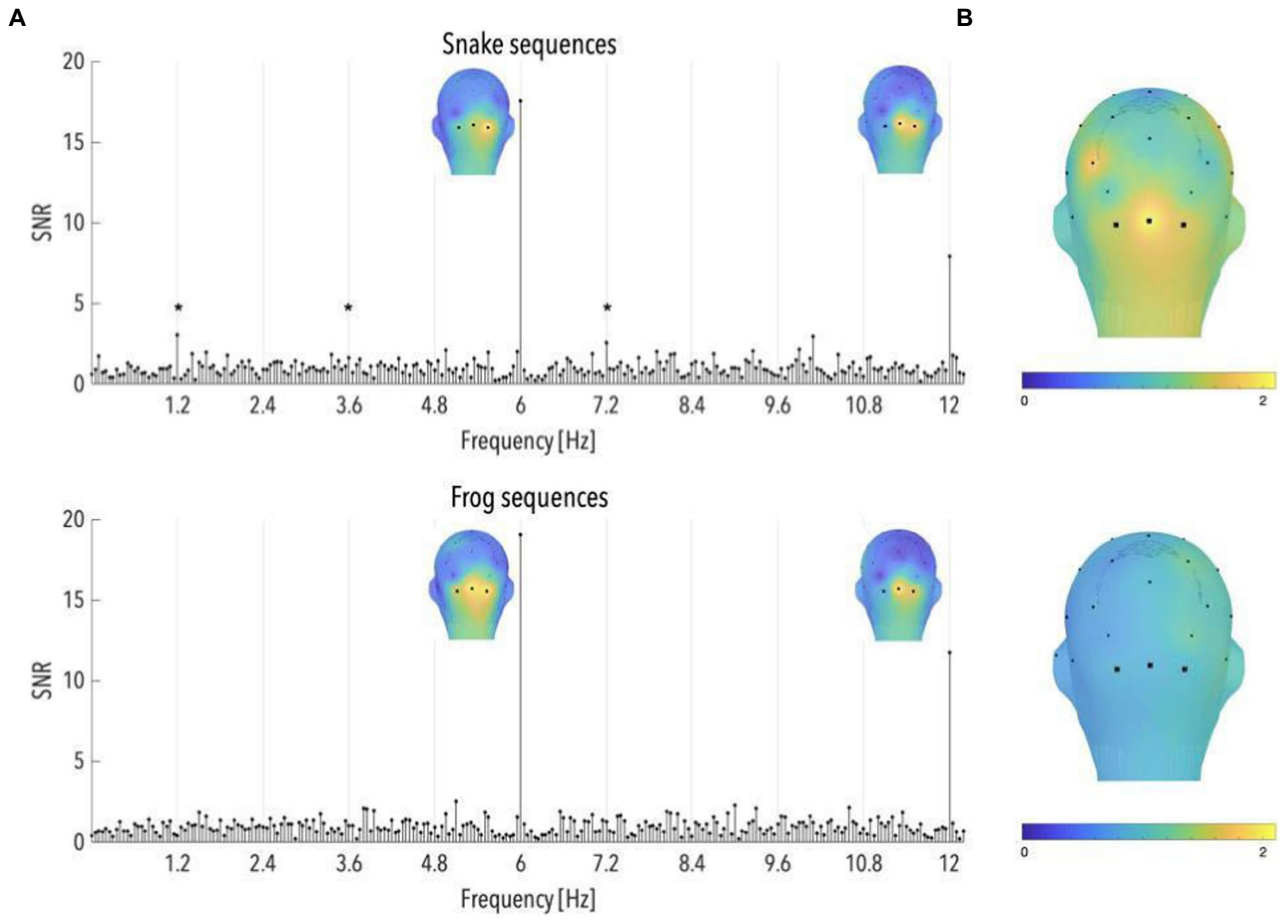
**(A)** Signal-to-noise ratio (SNR) spectra of category-selective and stimulation responses until 12 Hz, in the occipital region (data have been averaged across O1, Oz, and O2), and topographical maps of SNR over posterior scalp regions at stimulation frequencies, for snake and frog sequences (*n* = 87 and 91, respectively). The topographies at 6 and 12 Hz are shown on their individual maximal color scales. The asterisk indicates a significant discrimination response in the occipital region, of colorful snake pictures. **(B)** Topographical maps of SNR averaged on the first six harmonics of the category-selective response, for snake and frog sequences (upper and lower part, respectively).

Grand-averaged SNR spectra performed on the selected sequences showed clear responses at the stimulation frequencies (6 and 12 Hz), which attested for the successful synchronization of the infants’ visual system to the fast presentation of animal pictures. For both snake and frog sequences, these responses were characterized by a medial occipital topography (mean SNRs over the occipital electrodes in the snake sequences at 6 and 12 Hz = 17.57 and 7.88, *z*-scores = 33.08 and 11.91; mean SNRs in the frog sequences = 19.08 and 11.74, *z*-scores = 40.37 and 24.81; see Figure 2A).

The comparison between the z-scores associated with the occipital SNR values in snake and frog sequences at the stimulation frequencies did not reveal any significant difference (both *p* > 0.45).

Correlational analyses revealed no significant relationship between the infants’ age and the responses at the stimulation frequencies, neither for snake or frog sequences (at 6 Hz: *r_s_* = −0.003, *p* = 0.990 and *r_s_* = −0.163, *p* = 0.468; at 12 Hz: *r_s_* = 0.169, *p* = 0.451 and *r_s_* = −0.167, *p* = 0.459).

### Category-selective responses (1.2 Hz and harmonics)

Significant responses to snake pictures were observed at 1.2, 3.6, and 7.2 Hz in the occipital region (mean SNRs = 3.04, 1.59 and 2.56; *ps* < 0.05, z-scores = 2.35, 2.23 and 3.40; see Figure 2A). The same was however not true for frog sequences, at any of the harmonics before 12 Hz (*ps* > 0.05; see Figure 2A). As a consequence, the averaged brain response across the first 6 harmonics (i.e., until the last significant harmonic for either frog or snake sequences; see Methods) revealed a significant brain signal, in the occipital region, in response to snake (SNR = 1.78, *p* < 0.001; see Figure 2B) but not frog pictures (SNR = 0.91, *p* > 0.70; see Figure 2B). Importantly, the z-score associated with the SNR value in the occipital region averaged across the first 6 harmonics was significantly higher for snakes compared to frogs (*p* < 0.01).

These results thus replicate Bertels et al. (2020)‘s findings of a specific response in the infant brain to pictures of snakes in their natural background, in comparison to similarly colorful and unfamiliar but threat-irrelevant animals.

**Figure 3:**
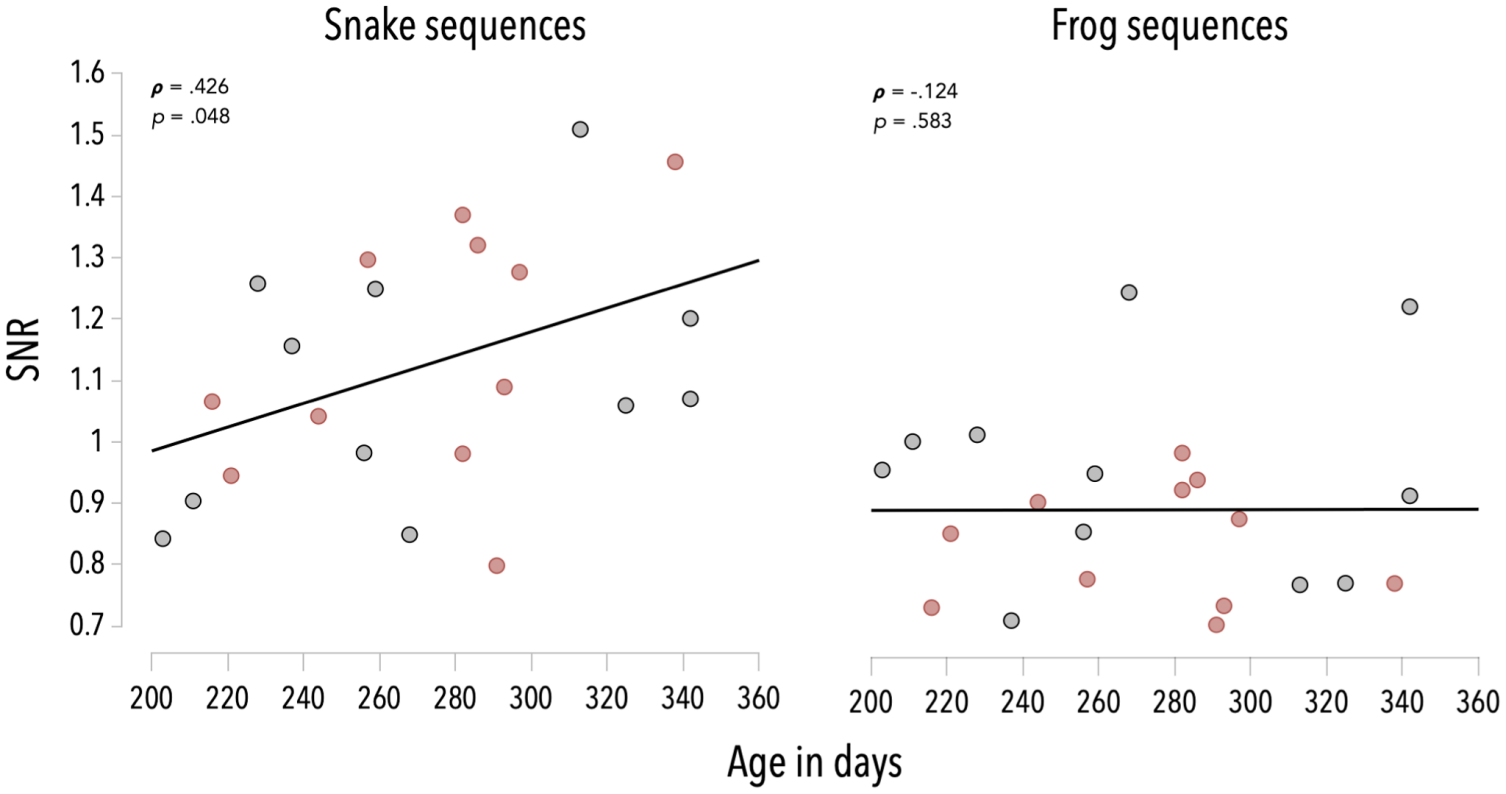
Associations between infants’ age and SNR values averaged on the first six harmonics of the category-selective response, for snake and frog sequences. Red dots refer to infants in the color group, grey dots refer to infants in the grayscale group.

We estimated the correlations between the infants’ age and the category-selective response averaged on the first six harmonics, for snake and frog sequences (see Figure 3). These analyses revealed that the older the infant, the higher the response to snakes (*r_s_* = 0.426, *p* = 0.048). This association was not significant for frog sequences (*r_s_* = −0.124, *p* = 0.583).

### Effect of color on category-selective responses

We further analyzed data separately for the color and grayscale infant groups to examine the role of color on the snake responses.

**Figure 4:**
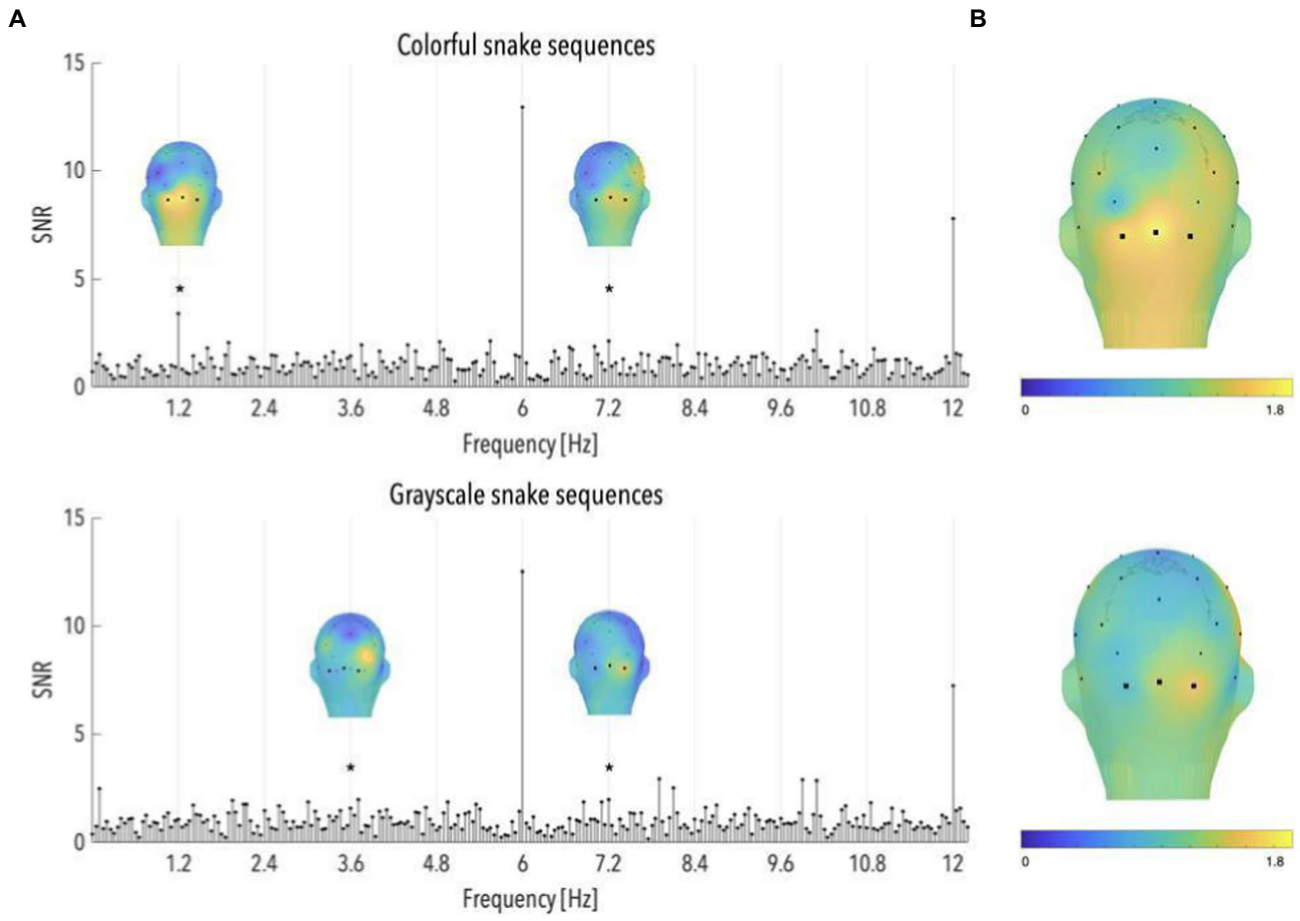
**(A)** Signal-to-noise ratio (SNR) spectra of category-selective and stimulation responses until 12 Hz, in the occipital region (data have been averaged across O1, Oz, and O2), and topographical maps of SNR over posterior scalp regions at significant category-selective frequencies, for colorful and grayscale snake sequences (*n* = 55 and 32, respectively). The topographies at the harmonics are shown on their individual maximal color scales. The asterisk indicates a significant discrimination response in the occipital region, of colorful and grayscale snake pictures. **(B)** Topographical maps of SNR averaged on the first six harmonics of the category-selective response, for colorful and grayscale snake sequences (upper and lower part, respectively).

Analyses of colorful snake sequences revealed significant brain responses at 1.2 and 7.2 Hz (mean occipital SNRs = 3.39 and 2.10; *ps* < 0.03, *z*-scores = 3.69 and 2.02; see Figure 4A), and a significant averaged response across the first 6 harmonics (SNR = 1.74, *p* < 0.001; see Figure 4B). Grayscale snake sequences led to a similar pattern with significant occipital responses observed at 3.6 and 7.2 Hz (mean SNRs = 1.57 and 1.98; *ps* < 0.03, *z*-scores = 2.11 and 2.01; see Figure 4A), as well as a significant averaged response across the first 6 harmonics (SNR = 1.32, *p* < 0.05; see Figure 4B).

To quantify differences between colorful and grayscale snake-specific responses, we compared the z-scores associated with the occipital SNR values in colorful and grayscale snake sequences. As the number of snake sequences considered in each group differed (55 in the color vs. 32 in the grayscale group), we randomly discarded 23 colorful sequences to ensure comparability of SNR measures between conditions. Analyses were run on 20 random selections of sequences to ensure the observed pattern did not depend on the selection of colorful sequences. No significant difference emerged between SNR values averaged across the first six harmonics, nor at 3.6 and 7.2 Hz (*p*s > 0.10). Comparisons at 1.2 Hz revealed higher responses to colorful than to grayscale snake pictures but the significance of that difference depended on the selection of sequences, with about half the selections leading to a significant difference.

Associations between age and category-selective responses—though positive—did not reach significance when considering separately colorful and grayscale snake sequences (*r_s_* = 0.405, *p* = 0.216 and *r_s_* = 0.337, *p* = 0.311; see Figure 3).

Of note, no significant frog-selective responses were observed, either in the color or in the grayscale group (*p*s > 0.05; see Supplementary Figure S2). No association was observed between the infants’ age and the non-significant category-selective responses to colorful or grayscale frogs (both *r_s_* < −0.10, *p* > 0.80).

## Discussion

Humans are remarkable snake detectors, and this ability would have evolved from the vital need to react adequately in the presence of these ancient, major predators. We recently demonstrated that this evolved predisposition to rapidly detect snakes is brain-anchored and already effective in infants (Bertels et al., 2020). As a matter of fact, snake pictures elicited specific neural responses in the infant occipital brain regions compared to similarly unfamiliar and elongated but uncoiled animals. However, in Bertels et al. (2020) the coiled shape of snakes was blended with other possibly critical visual features, notably their coloration. Yet, coloration has been shown to contribute to the rapid processing of faces and natural scenes (Goffaux et al., 2005; Or et al., 2019), and acts as a powerful aposematic signal (Stevens and Ruxton, 2012). Here, we aimed at clarifying the role of color information in the infants’ brain responses to snakes by contrasting the effects of colorful vs. grayscale images.

While we replicated Bertels et al. (2020)‘s findings of a selective neural response to snakes, the results do not provide convincing evidence that color information plays a critical role in this response. As a matter of fact, snake-specific responses were not only observed when snakes were depicted in color, but also when grayscale versions of these reptiles were presented to the infants. These results further support that the shape of snakes is a crucial factor in their rapid detection (Lobue and Deloache, 2011). They mirror previous findings in children, adults, and non-human primates that color is not necessary to detect snakes faster than non-snake controls (Shibasaki and Kawai, 2009; Masataka et al., 2010; Hayakawa et al., 2011; Lobue and Deloache, 2011; Kawai and Koda, 2016). Using neurophysiological measures, our results extend these findings to human infants below 1 year of age.

In our study, responses were however stronger—though not significantly so—when infants were exposed to colorful rather than grayscale snakes. Color information could therefore contribute, albeit slightly, to the rapid categorization of snakes, as it does for faces that are also highly relevant stimuli for the survival of the species (Or et al., 2019). This observation echoes recent findings that trichromat humans—as are most representatives of our species by 3 months (Teller, 1998)—outperform dichromats in detecting predators (Pessoa et al., 2014; de Moraes et al., 2021). These findings have been taken as evidence in favor of the *trichromatic advantage for predator detection hypothesis* (Pessoa et al., 2014) according to which trichromatic color vision evolved in humans and closely related primates due to its evolutionary advantage in detecting predators. In this view, predation pressure, as well as the urge to detect food (Sumner and Mollon, 2003) and mate (Changizi et al., 2006), acted as selective factors to favor trichromacy in humans and Old World monkeys. This hypothesis and its supporting evidence further emphasize the role that color, and color vision more generally, plays in predator detection.

Color does not have a decisive influence on the detection of frogs either. As a matter of fact, we did not observe any frog-selective response whether frogs were presented in color or in black and white. Of note, the absence of a significant response to colorful frogs contrasts with previous findings by Bertels et al. (2020). Yet the number of participants and frog sequences included in both studies is comparable (*n* = 22 vs. 26, and *n* = 91 vs. 109). We hypothesize this result to rise from the fact that the neural responses to frogs are less robust than those to snakes, in infants.

While color only slightly influenced snake detection in our study, and had no effect on neural responses to frogs and other animals, it nevertheless largely impacted the overall amount of attention that infants devoted to flickering stimuli. In fact, infants attentively looked at almost twice as many colored sequences as grayscale ones. This result is in line with studies showing that color stimuli grab more attention than grayscale stimuli (Zhu et al., 2013) and that, from 4 months of age, infants prefer colorful to grayscale exemplars of the same stimuli (Spears, 1966). Color also eases figure-ground segmentation (Delorme et al., 1999; Gegenfurtner and Rieger, 2000), which probably made our pictures more interesting to look at when presented in their color than grayscale versions.

Remarkably, the neural categorical responses to colorful and grayscale snakes increased with age during the second half of the first year, with older infants showing stronger responses to snakes. This finding could be attributed to the general maturation of the visual system. Indeed, even though infants discriminate black-and-white patterns from their first days of life (Fantz and Miranda, 1975), detect complex visual stimuli such as animal or human faces in grayscale image arrays already at 3 months (Simpson et al., 2019), and have an advanced perception of color (Skelton et al., 2022) and visual acuity close to adult levels at 8 months (Courage and Adams, 1990), visual discrimination takes years to refine (Skelton et al., 2022). Obviously, infants also gained more experience during that period, notably with animals, being progressively able to recognize them and discriminate between species. However, if the growing experience was the main cause for that increased response to snakes, we should have observed a similar increase in the brain responses to frogs, which we did not. Rather, there seems to be a specific development in the ability to detect snakes, although throughout the age range tested infants remain naïve about their potential danger. We would therefore argue that the observed improvement in the ability to detect snakes between 6 and 11 months of age relates to the specific development of the visual system with respect to evolutionary-relevant shapes embedded in complex backgrounds, rather than to increased experience with animals, in general.

Overall, this study reveals that color information is not necessary for infants to swiftly detect snakes in natural scenes. Together with previous studies on infants and older individuals, it corroborates that the coiled shape of snakes is the critical feature driving fast response to snakes in primates. It also provides evidence that snake-specific neural responses increase as the visual system matures. Future studies should investigate larger age ranges, from birth on, and explore, using stimuli adapted to the infants’ visual abilities (e.g., contrasting stimuli with saturated colors and a background mask), how these responses evolve with developing visual systems, growing life experience, accumulated learning and possible fear experience.

## Data availability statement

The datasets generated and analyzed during the current study are available upon reasonable request from the corresponding author.

## Ethics statement

The studies involving human participants were reviewed and approved by CUB Hôpital Erasme Ethics Committee. Written informed consent to participate in this study was provided by the participants’ legal guardian/next of kin.

## Author contributions

JB, AdH, and AD conceived the study. AdH implemented the experiment. JB and AdH performed the study. JB and MB analyzed the data. JB wrote the manuscript. AD, MB, AdH, and AC edited the manuscript. All authors contributed to the article and approved the submitted version.

## Funding

This work was supported by a FRS–FNRS grant (F.4524.10). JB and AdH were Post-Doctoral Researchers of the Fonds de la Recherche Scientifique-FNRS.

## Conflict of interest

The authors declare that the research was conducted in the absence of any commercial or financial relationships that could be construed as a potential conflict of interest.

## Publisher’s note

All claims expressed in this article are solely those of the authors and do not necessarily represent those of their affiliated organizations, or those of the publisher, the editors and the reviewers. Any product that may be evaluated in this article, or claim that may be made by its manufacturer, is not guaranteed or endorsed by the publisher.

## Footnotes

^1^https://calphotos.berkeley.edu/fauna

^2^http://letswave.org

## References

[ref1] Barry-Anwar R. Hadley H. Conte S. Keil A. Scott L. S. (2018). The developmental time course and topographic distribution of individual-level monkey face discrimination in the infant brain. Neuropsychologia 108, 25–31. doi: 10.1016/j.neuropsychologia.2017.11.019, PMID: 29157998

[ref2] Bertels J. Bayard C. Floccia C. Destrebecqz A. (2018). Rapid detection of snakes modulates spatial orienting in infancy. Int. J. Behav. Dev. 42, 381–387. doi: 10.1177/0165025417693955

[ref3] Bertels J. Bourguignon M. de Heering A. Chetail F. De Tiège X. Cleeremans A.. (2020). Snakes elicit specific neural responses in the human infant brain. Sci. Rep. 10:7443. doi: 10.1038/s41598-020-63619-y, PMID: 32366886 PMC7198620

[ref4] Changizi M. A. Zhang Q. Shimojo S. (2006). Bare skin, blood and the evolution of primate colour vision. Biol. Lett. 2, 217–221. doi: 10.1098/rsbl.2006.0440, PMID: 17148366 PMC1618887

[ref5] Courage M. L. Adams R. J. (1990). Visual acuity assessment from birth to three years using the acuity card procedure: cross-sectional and longitudinal samples. Am. J. Optom. Physiol. Optic 67, 713–718. doi: 10.1097/00006324-199009000-00011, PMID: 2234832

[ref6] de Heering A. Rossion B. (2015). Rapid categorization of natural face images in the infant right hemisphere. elife 4:e06564. doi: 10.7554/eLife.06564, PMID: 26032564 PMC4450157

[ref7] de Moraes P. Z. Diniz P. Spyrides M. H. C. Pessoa D. M. A. (2021). The effect of pelage, background, and distance on predator detection and the evolution of primate color vision. Am. J. Primatol. 83:e23230. doi: 10.1002/ajp.23230, PMID: 33475188

[ref8] Deloache J. S. Lobue V. (2009). The narrow fellow in the grass: human infants associate snakes and fear. Dev. Sci. 12, 201–207. doi: 10.1111/j.1467-7687.2008.00753.x, PMID: 19120429

[ref9] Delorme A. Richard G. Fabre-Thorpe M. (1999). Rapid processing of complex natural scenes: a role for the magnocellular visual pathways? Neurocomputing 26-27, 663–670. doi: 10.1016/s0925-2312(98)00158-1

[ref10] Fančovičová J. Prokop P. Szikhart M. Pazda A. (2020). Snake coloration does not influence children’s detection time. Hum. Dimens. Wildl. 25, 489–497. doi: 10.1080/10871209.2020.1758252

[ref11] Fantz R. L. Miranda S. B. (1975). Newborn infant attention to form of contour. Child Dev. 46, 224–228. doi: 10.2307/1128853, PMID: 1132272

[ref12] Gegenfurtner K. R. Rieger J. (2000). Sensory and cognitive contributions of color to the recognition of natural scenes. Curr. Biol. 10, 805–808. doi: 10.1016/S0960-9822(00)00563-7, PMID: 10898985

[ref13] Goffaux V. Jacques C. Mouraux A. Oliva A. Schyns P. Rossion B. (2005). Diagnostic colours contribute to the early stages of scene categorization: Behavioural and neurophysiological evidence. Vis. Cogn. 12, 878–892. doi: 10.1080/13506280444000562

[ref14] Gomes N. Soares S. C. Silva S. Silva C. F. (2018). Mind the snake: fear detection relies on low spatial frequencies. Emotion 18, 886–895. doi: 10.1037/emo0000391 29265840

[ref15] Hayakawa S. Kawai N. Masataka N. (2011). The influence of color on snake detection in visual search in human children. Sci. Rep. 1:1. doi: 10.1038/srep00080, PMID: 22355599 PMC3216567

[ref16] He H. Kubo K. Kawai N. (2014). Spiders do not evoke greater early posterior negativity in the event-related potential as snakes. Neuroreport 25, 1049–1053. doi: 10.1097/WNR.0000000000000227 25026534

[ref17] Heinrich S. P. (2010). Some thoughts on the interpretation of steady-state evoked potentials. Doc. Ophthalmol. 120, 205–214. doi: 10.1007/s10633-010-9212-7, PMID: 20101435

[ref18] Isbell L. A. (2009). The Fruit, the Tree, and the Serpent. Cambridge, MA: Harvard University Press.

[ref19] Isbell L. A. Etting S. F. (2017). Scales drive detection, attention, and memory of snakes in wild vervet monkeys (*Chlorocebus pygerythrus*). Primates 58, 121–129. doi: 10.1007/s10329-016-0562-y, PMID: 27517268

[ref20] Kawai N. (2019). The Fear of Snakes. The Science of the Mind. Berlin: Springer.

[ref21] Kawai N. Koda H. (2016). Japanese monkeys (*Macaca fuscata*) quickly detect snakes but not spiders: evolutionary origins of fear-relevant animals. J. Comp. Psychol. 130, 299–303. doi: 10.1037/com0000032, PMID: 27078076

[ref22] Liu-Shuang J. Norcia A. M. Rossion B. (2014). An objective index of individual face discrimination in the right occipito-temporal cortex by means of fast periodic oddball stimulation. Neuropsychologia 52, 57–72. doi: 10.1016/j.neuropsychologia.2013.10.022, PMID: 24200921

[ref23] Lobue V. DeLoache J. S. (2008). Detecting the snake in the grass: attention to fear-relevant stimuli by adults and young children. Psychol. Sci. 19, 284–289. doi: 10.1111/j.1467-9280.2008.02081.x 18315802

[ref24] LoBue V. DeLoache J. S. (2010). Superior detection of threat-relevant stimuli in infancy. Dev. Sci. 13, 221–228. doi: 10.1111/j.1467-7687.2009.00872.x, PMID: 20121878

[ref25] Lobue V. Deloache J. S. (2011). What’s so special about slithering serpents? Children and adults rapidly detect snakes based on their simple features. Vis. Cogn. 19, 129–143. doi: 10.1080/13506285.2010.522216

[ref26] Masataka N. Hayakawa S. Kawai N. (2010). Human young children as well as adults demonstrate “superior” rapid snake detection when typical striking posture is displayed by the snake. PLoS One 5:e15122. doi: 10.1371/journal.pone.0015122, PMID: 21152050 PMC2994910

[ref27] Nichols T. E. Holmes A. P. (2002). Nonparametric permutation tests for functional neuroimaging: a primer with examples. Hum. Brain Mapp. 15, 1–25. doi: 10.1002/hbm.1058, PMID: 11747097 PMC6871862

[ref28] Norcia A. M. Appelbaum L. G. Ales J. M. Cottereau B. R. Rossion B. (2015). The steady-state visual evoked potential in vision research: a review. J. Vis. 15:4. doi: 10.1167/15.6.4, PMID: 26024451 PMC4581566

[ref29] Oliva A. Schyns P. G. (2000). Diagnostic colors mediate scene recognition. Cogn. Psychol. 41, 176–210. doi: 10.1006/cogp.1999.0728, PMID: 10968925

[ref30] Or C. C.-F. Retter T. L. Rossion B. (2019). The contribution of color information to rapid face categorization in natural scenes. J. Vis. 19:20. doi: 10.1167/19.5.20, PMID: 31112241

[ref31] Pessoa D. M. A. Maia R. de Albuquerque Ajuz R. C. De Moraes P. Z. P. M. R. Spyrides M. H. C. Pessoa V. F. (2014). The adaptive value of primate color vision for predator detection. Am. J. Primatol. 76, 721–729. doi: 10.1002/ajp.22264, PMID: 24535839

[ref32] Peykarjou S. (2022). Frequency tagging with infants: the visual oddball paradigm. Front. Psychol. 13:1015611. doi: 10.3389/fpsyg.2022.1015611, PMID: 36425830 PMC9679632

[ref33] Peykarjou S. Hoehl S. Pauen S. Rossion B. (2017). Rapid categorization of human and ape faces in 9-month-old infants revealed by fast periodic visual stimulation. Sci. Rep. 7:12526. doi: 10.1038/s41598-017-12760-2, PMID: 28970508 PMC5624891

[ref34] Peykarjou S. Langeloh M. Baccolo E. Rossion B. Pauen S. (2022). Superior neural individuation of mother’s than stanger’s faces by five months of age. Cortex 155, 264–276. doi: 10.1016/j.cortex.2022.07.011, PMID: 36044787

[ref35] Prokop P. Fančovičová J. Kučerová A. (2018). Aposematic colouration does not explain fear of snakes in humans. J. Ethol. 36, 35–41. doi: 10.1007/s10164-017-0533-9

[ref36] Rekow D. Baudouin J.-Y. Poncet F. Damon F. Durand K. Schaal P. (2021). Odor-driven face-like categorization in the human infant brain. PNAS 118:e2014979118. doi: 10.1073/pnas.2014979118, PMID: 34001601 PMC8166123

[ref37] Retter T. L. Rossion B. Schiltz C. (2021). Harmonic amplitude summation for frequency-tagging analysis. J. Cogn. Neurosci. 33, 2372–2393. doi: 10.1162/jocn_a_01763, PMID: 34272961

[ref01] Rossion B. Retter T. L. Liu-Shang J. (2020). Understanding human individuation of unfamiliar faces with oddball fast periodic visual stimulation and electroencephalography. Eur. J. Neurosci. 52, 4283–4344. 32542962 10.1111/ejn.14865

[ref38] Ruxton G. D. Sherratt T. N. Speed M. P. (2004). Avoiding Attack. Oxford: Oxford University Press.

[ref39] Shibasaki M. Kawai N. (2009). Rapid detection of snakes by Japanese monkeys (*Macaca fuscata*): an evolutionarily predisposed visual system. J. Comp. Psychol. 123, 131–135. doi: 10.1037/a0015095, PMID: 19450020

[ref02] Simpson E. A. Maylott S. E. Leonard K. Lazo R. J. Jakobsen K. V. (2019). Face detection in infants and adults: Effects of orientation and color. J. Exp. Child Psychol. 186, 17–32. 31185365 10.1016/j.jecp.2019.05.001

[ref40] Skelton A. E. Maule J. Franklin A. (2022). Infant color perception: insight into perceptual development. Child Dev. Perspect. 16, 90–95. doi: 10.1111/cdep.12447, PMID: 35915666 PMC9314692

[ref41] Soares S. C. Lindström B. Esteves F. Ohman A. (2014). The hidden Snake in the grass: superior detection of snakes in challenging attentional conditions. PLoS One 9:e114724. doi: 10.1371/journal.pone.0114724, PMID: 25493937 PMC4262429

[ref42] Souchet J. Aubret F. (2016). Revisiting the fear of snakes in children: the role of aposematic signalling. Sci. Rep. 6:37619. doi: 10.1038/srep37619, PMID: 27886218 PMC5122844

[ref43] Spears W. C. (1966). Visual preference in the four-month old infant. Psychon. Sci. 4, 237–238. doi: 10.3758/BF03342270

[ref44] Stevens M. Ruxton G. D. (2012). Linking the evolution and form of warning coloration in nature. Proc. Biol. Sci. 279, 417–426. doi: 10.1098/rspb.2011.1932, PMID: 22113031 PMC3234570

[ref45] Sumner P. Mollon J. D. (2003). Colors of primate pelage and skin: objective assessment of conspicuousness. Am. J. Primatol. 59, 67–91. doi: 10.1002/ajp.10066, PMID: 12619048

[ref46] Teller D. Y. (1998). Spatial and temporal aspects of infant color vision. Vis. Res. 38, 3275–3282. doi: 10.1016/S0042-6989(97)00468-9, PMID: 9893838

[ref47] Van Le Q. Isbell L. A. Matsumoto J. Nguyen M. Hori E. Maior R. S.. (2013). Pulvinar neurons reveal neurobiological evidence of past selection for rapid detection of snakes. Proc. Natl. Acad. Sci. U. S. A. 110, 19000–19005. doi: 10.1073/pnas.1312648110, PMID: 24167268 PMC3839741

[ref48] Van Strien J. W. Christiaans G. Franken I. H. A. Huijding J. (2016). Curvilinear shapes and the snake detection hypothesis: an ERP study. Psychophysiology 53, 252–257. doi: 10.1111/psyp.12564, PMID: 26481589

[ref49] Van Strien J. W. Isbell L. A. (2017). Snake scales, partial exposure, and the Snake detection theory: a human event-related potentials study. Sci. Rep. 7:46331. doi: 10.1038/srep46331, PMID: 28387376 PMC5384215

[ref50] Zhu W. Drewes J. Gegenfurtner K. R. (2013). Animal detection in natural images: effects of color and image database. PLoS One 8:e75816. doi: 10.1371/journal.pone.0075816, PMID: 24130744 PMC3794973

