# Supplementary figures and images for: What determines the neural response to snakes in the infant brain? A systematic comparison of color and grayscale stimuli
Source: Front Psychol. 2023 Mar 13;14:1027872. doi: 10.3389/fpsyg.2023.1027872 (PMC10040846; doi:10.3389/fpsyg.2023.1027872)

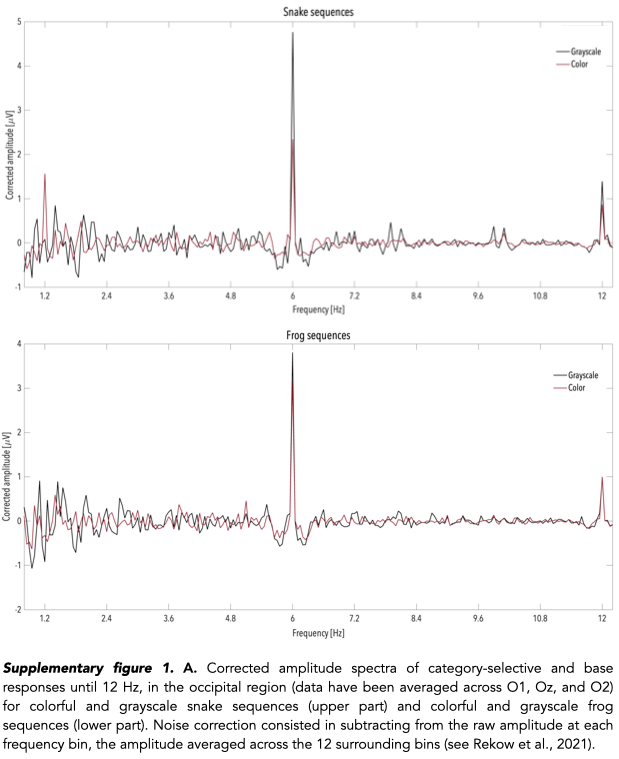

Supplement: Supplementary file 1 [file Image_1.tiff]

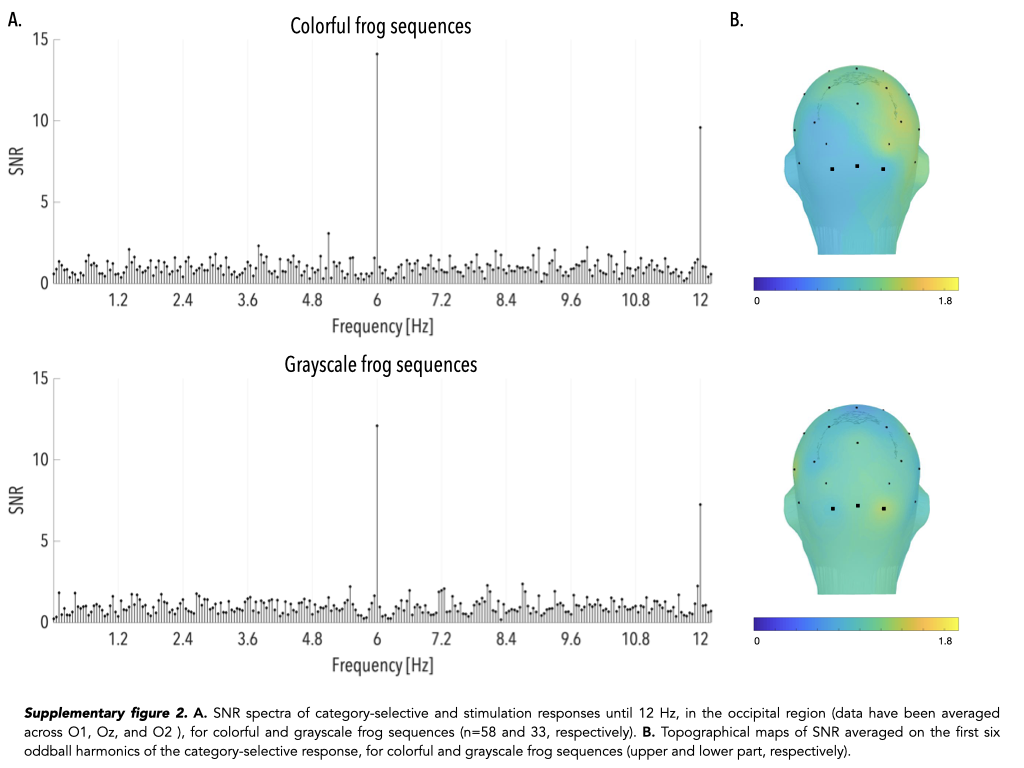

Supplement: Supplementary file 2 [file Image_2.tiff]
